# A global assessment of the mixed layer in coastal sediments and implications for carbon storage

**DOI:** 10.1038/s41467-022-32650-0

**Published:** 2022-08-20

**Authors:** Shasha Song, Isaac R. Santos, Huaming Yu, Faming Wang, William C. Burnett, Thomas S. Bianchi, Junyu Dong, Ergang Lian, Bin Zhao, Lawrence Mayer, Qingzhen Yao, Zhigang Yu, Bochao Xu

**Affiliations:** 1 Frontiers Science Center for Deep Ocean Multispheres and Earth System, Key Laboratory of Marine Chemistry Theory and Technology, Ministry of Education, Ocean University of China, Qingdao, 266100 P. R. China; 2 Laboratory for Marine Ecology and Environmental Science, Qingdao National Laboratory for Marine Science and Technology, Qingdao, 266100 P. R. China; 3 College of Chemistry and Chemical Engineering, Ocean University of China, Qingdao, 266100 P. R. China; 4 Department of Marine Sciences, University of Gothenburg, Göteborg, 40530 Sweden; 5 National Marine Science Centre, School of Environment, Science and Engineering, Southern Cross University, Coffs Harbour, NSW 2450 Australia; 6 College of Oceanic and Atmospheric Sciences, Ocean University of China, Qingdao, 266100 P. R. China; 7 Sanya Oceanographic Institution, Ocean University of China, Sanya, 572000 P. R. China; 8 Xiaoliang Research Station for Tropical Coastal Ecosystems, Key Laboratory of Vegetation Restoration and Management of Degraded Ecosystems, and the CAS engineering Laboratory for Ecological Restoration of Island and Coastal Ecosystems, South China Botanical Garden, Chinese Academy of Sciences, Guangzhou, 510650 P.R. China; 9 Department of Earth, Ocean, and Atmospheric Science, Florida State University, Tallahassee, FL 32306 USA; 10 Department of Geological Sciences, University of Florida, Gainesville, FL 32611-2120 USA; 11 School of Computer Science and Technology, Ocean University of China, Qingdao, 266100 P. R. China; 12 State Key Laboratory of Marine Geology, Tongji University, Shanghai, 200092 P. R. China; 13 School of Marine Sciences, University of Maine, Walpole, Maine, ME 04573 USA

**Keywords:** Marine chemistry, Biogeochemistry

## Abstract

The sediment-water interface in the coastal ocean is a highly dynamic zone controlling biogeochemical fluxes of greenhouse gases, nutrients, and metals. Processes in the sediment mixed layer (SML) control the transfer and reactivity of both particulate and dissolved matter in coastal interfaces. Here we map the global distribution of the coastal SML based on excess ^210^Pb (^210^Pb_ex_) profiles and then use a neural network model to upscale these observations. We show that highly dynamic regions such as large estuaries have thicker SMLs than most oceanic sediments. Organic carbon preservation and SMLs are inversely related as mixing stimulates oxidation in sediments which enhances organic matter decomposition. Sites with SML thickness >60 cm usually have lower organic carbon accumulation rates (<50 g C m^−2^ yr^−1^) and total organic carbon/specific surface area ratios (<0.4 mg m^−2^). Our global scale observations reveal that reworking can accelerate organic matter degradation and reduce carbon storage in coastal sediments.

## Abstract (web summary)

The authors map the global distribution of the mixed layer in coastal ocean sediments, based on a neural network model. These observations reveal that mixing can accelerate organic matter degradation and reduce carbon storage in the coastal ocean.

## Introduction

Coastal sediments record detailed historical changes of land-use and climate, which can impact source-to-sink particle dynamics across the land-ocean boundary^1^. These sediment records can be altered by physical and/or biological mixing which can modify sedimentary structures and obscure record interpretations^2^. Constraining the thickness and location of the sediment mixed layer (SML) is essential for resolving key pathways in marine biogeochemical cycles^3,4^. For instance, the SML is an important driver of the exchange of nutrients, organic carbon, redox-sensitive elements and greenhouse gases between the seafloor and the overlying seawater^5,6^.

Quantifying the thickness of the SML is challenging, particularly in highly dynamic coastal sediments. Atmospherically derived ^210^Pb (half-life of 22.3 years) is a natural “tracer” for sediments accumulating over time. Pioneering work using ^210^Pb for dating marine sediments^7^, established chronologies in estuarine and coastal sediments^8–10^. In rapidly changing coastal environments, sediments are commonly remobilized as stationary fluid muds, and/or resuspended and laterally transported as dense suspensions^11,12^. Particle reworking and remobilization can result in loss of chronological information^13–15^ and is highly heterogeneous spatially and temporally. Thus, the distribution and thickness of the SML are important to improve global flux estimates of dissolved and particulate constituents of key biogeochemical cycles in Earth System Models^16^.

Neural networks have emerged as a powerful tool to resolve complex spatial and temporal patterns that are common in large datasets in the geosciences^17,18^. Some examples of neural networks applications in the earth sciences include: hydrology, including flooding forecasts, and water quality modeling^19,20^; geophysics/geomorphology, including earthquake predictions, and simulating land-use change^21,22^; and atmospheric sciences, by modeling cloud formation and temperatures^23,24^. Recently, machine learning (e.g., K-nearest neighbor, random forest and neural networks) has been applied to resolve important questions in oceanography, including the global distribution of seafloor total organic carbon, benthic properties, sediment porosity/density and sediment accumulation rates^25–30^.

While the SML has been assessed on local and regional scales^12,15^, global-scale datasets remain sparse. A tracer-identified surface mixed-depth global mean value of 9.8 ± 4.5 cm was obtained in the 1990’s^31,32^ and later updated to 5.75 ± 5.67 cm^33^. Nevertheless, there remains a significant gap in our knowledge of the distribution patterns of SMLs in the global coastal ocean. Hence, we posit that a more accurate estimate of SMLs is needed for better incorporation into global biogeochemical ocean models.

Here, we define the SML as the sediment thickness captured by ^210^Pb_ex_ profiles that has been reworked over time scales of months^34,35^. We estimated SML thicknesses in the global coastal ocean by compiling data from 742 globally distributed sediment cores. First, we evaluated the spatial patterns and drivers of sediment mixing. We then used a neural network model to estimate the thickness of SMLs in the entire global ocean shallower than 200 m, linked it to organic carbon burial in marine sediments. This global assessment of coastal SMLs improves our ability to estimate and interpret the burial capacity and remineralization of organic matter and nutrients in ocean sediments.

## Results and discussion

### Estimating the global coastal sediment mixed layer

**Fig. 1:**
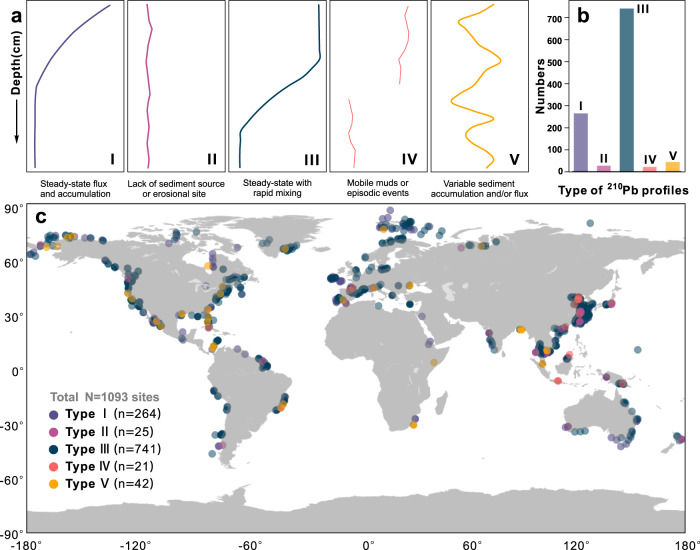
Profiles of excess ^210^Pb (^210^Pb_ex_) in global coastal ocean. **a** Sketches of five sedimentary ^210^Pb_ex_ patterns in coastal areas based on downcore ^210^Pb_ex_ profiles; the *x*-axis represents ^210^Pb_ex_ activities. The frequency (**b**) and global distribution (**c**) of five types of ^210^Pb_ex_ profiles in the global coastal ocean is based on data from 1093 cores collected from the literature (Supplementary Data 1). Map of panel c generated with python3-mpltoolkits.basemap (version 1.2.1, https://matplotlib.org/basemap/).

Profiles of excess ^210^Pb (^210^Pb_ex_) in coastal shelf and estuarine sediments, devoid of sediment mixing, typically reflect exponential decay with depth^1^. Vertical ^210^Pb_ex_ profiles have previously been divided into eight types to cover multiple mixing possibilities^12^. Here, we simplified this classification into five common types (Fig. 1a). A Type I profile is produced by constant sediment accumulation under steady-state conditions. In contrast, Type II profiles show no excess ^210^Pb activities with depth, reflective of scoured, eroded areas with exposures of once deeply buried material^34^. These two profiles are produced by sediment deposition in non-reworked settings; the other three types of ^210^Pb_ex_ profiles reflect disturbances related to sediment mixing (physical and/or biological). A Type III profile reflects constant ^210^Pb_ex_ activities along the upper layers of the core overlaying an exponentially decaying trend, usually attributed to mixing from sediment resuspension or reworking very recently (months to years)^35,36^. A Type IV profile also reveals an intense reworked surficial layer during an episodic disturbance event such as a storm, leading to deep homogenous ^210^Pb_ex_ activities in the upper section, overlying sediments with no excess ^210^Pb. The Type V profile can be used as evidence of repetitive reworking and deposition in a highly reworked mud stratigraphy^37^. The criteria used here in defining the thickness of the SML is based on the upper homogenized layer of ^210^Pb_ex_ in Type III and Type IV profiles, indicative of mud layers reworked on a timescale of months. Since any exclusion of other ^210^Pb_ex_ profile types can result in an underestimation of mixing depths. Thus, our use of Type III and Type IV profiles represents a conservative estimate of SML thicknesses on a global scale. Furthermore, it is possible that an exponential decrease in the upper section of a Type I profile could originate from slow/deep bioturbation. This kind of reworking is indistinguishable from profiles originating from sediment accumulation and/or a combination of sediment accumulation and bioturbation.

We compiled 1093 published ^210^Pb_ex_ profiles from the global continental shelf (see Methods and Supplementary Data 1). The geographic distribution of ^210^Pb_ex_ profile types of all 1093 sites (Fig. 1b, c) revealed a dominance of Type III, followed by Type I. Studies that used gravity cores to define SML were not applied in our modeling (except for the Amazon Shelf), because gravity cores are well-known to create a bow wave artifact that disrupts near-surface sediments^38^. However, the significant greater thicknesses of the SML in the Amazon Estuary made it difficult to obtain a complete ^210^Pb_ex_ profile when using short box-cores (<1 m). So, gravity-core data were included when analyzing SMLs in Amazon Shelf sediments. Overall, 742 cores had complete ^210^Pb_ex_ profiles and recognizable SMLs to be used in our model.

**Fig. 2:**
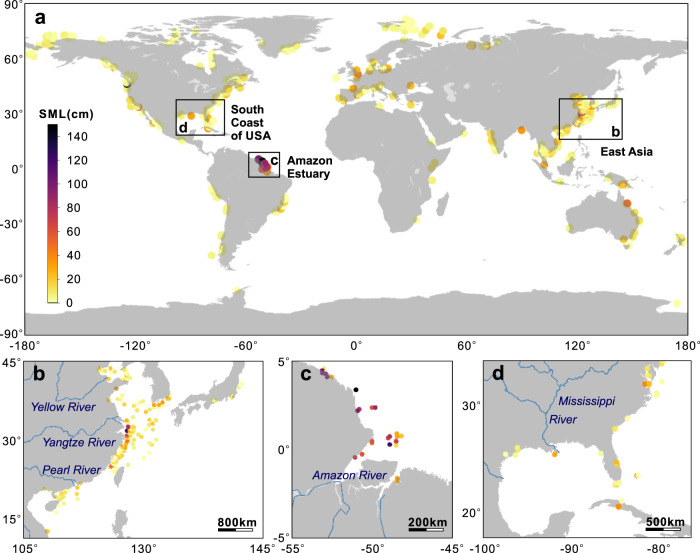
The thickness of the sediment mixed layer (SML) estimated by excess ^210^Pb (^210^Pb_ex_) profiles. **a** The global coastal ocean with 742 sites; **b** The coastal ocean of East Asia; **c** The Amazon Estuary; and **d** Gulf of Mexico. Maps generated with python3-mpltoolkits.basemap (version 1.2.1, https://matplotlib.org/basemap/).

The overall thickness of SMLs in the coastal ocean proved to be very heterogeneously distributed; ~47% of the SMLs were >5 cm, 36% had SMLs ranging from 5–20 cm, and only 3% had SMLs thicker than 30 cm (Fig. 2a). Thick SMLs primarily occurred in large-river delta-front estuaries (LDEs). Deep mixing could be viewed as inconsistent with the notion that these sediments are potential “recorders” of past natural and anthropogenic changes^39^. However, thick SMLs demonstrate the heterogeneity of depositional environments in LDEs, and the need for careful site selection of sampling locations for paleo-reconstruction work, which has proven to be successful in some LDEs (e.g., Mississippi River)^40,41^. These LDEs include the Yangtze (SML usually >20 cm and reaching m>100 cm locally, Fig. 2b)^12^, Amazon (usually >100 cm, Fig. 2c)^42^, and Ganges. SMLs in Asia are thicker, with thicknesses of 10–0 cm estimated to be 23% of the total (Supplementary Fig. 1). This finding is not surprising since Asia has the largest number of great rivers in the world^43^. In contrast, much thinner SMLs (0–5 cm, 35%) were found in North America away from large-river sources. In some enclosed marginal seas such as the Gulf of Mexico (Fig. 2d), Gulf of California and the Baltic Sea, the SML thicknesses are also significant, reaching up to 40 cm. Areas with negligible SMLs are mainly found in the coastal ocean at high latitudes.

### Drivers of SML

The SML thickness is controlled by (1) environmental factors such as precipitation, temperature, and water depth; (2) physical factors including winds, tides, waves, and bottom stress; (3) biological activities including feeding and burrowing; and (4) sediment sources. We identified 12 influencing factors (Methods) and analyzed their relationship to SMLs (Supplementary Fig. 2). The strongest correlation coefficients, in rank order, were river discharge (0.58), bottom stress (0.52), total suspended matter (0.35), primary productivity (0.20), and water depth (0.16). Correlations between SML and other potential drivers such as sediment accumulation rate, relative sea level change, tropical cyclone frequency and mean annual precipitation were non-significant. Bottom stress alone predicts a tight response of SML thickness with a range of 2 m (Supplementary Fig. 3a). Removing values with bottom stress >1 Pa shows that primary productivity (implying food-driven bioturbation) also predicts SML thickness (Supplementary Fig. 3b), with values consistent with depths mixed by animals^32^. Thus, we infer that physical forces account for the greatest sediment disturbance in coastal oceans, but that bioturbation is a strong control in regions with little physical mixing. Shallow systems with large sediment sources (such as deltas) usually have higher SML thicknesses due to stronger hydrodynamic forcing and larger watersheds with more extensive and varied human disturbances.

We incorporated these five strongest SML drivers into our neural network model to predict global SMLs (see Methods section). Since it is well established that SMLs largely occur in fine-grained deposits (e.g., both organic and inorganic particles)^44^, the ^210^Pb_ex_ profiles used here are almost exclusively from muddy deposits.

### Global upscaling with a neural network model

**Fig. 3:**
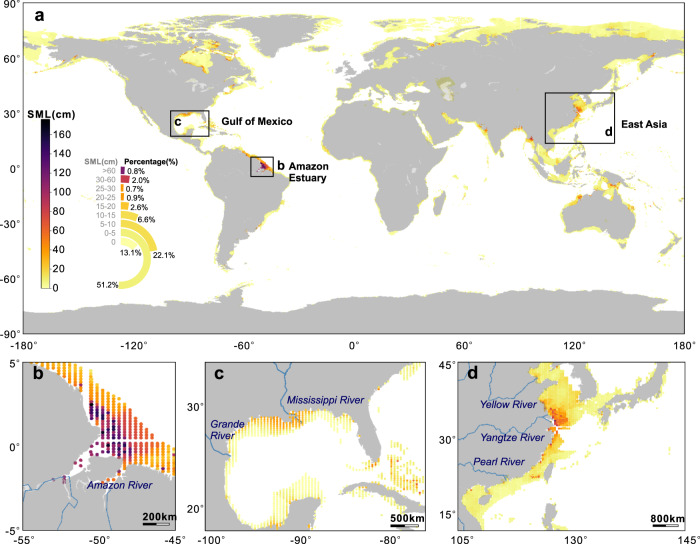
Simulation of sediment mixed layer (SML) thicknesses. **a** The global coastal ocean; **b** The Amazon Estuary; **c** Gulf of Mexico; and **d** The coastal ocean of East Asia. Maps generated with python3-mpltoolkits.basemap (version 1.2.1, https://matplotlib.org/basemap/).

Our neural network simulation of SMLs in the global coastal ocean with water depths shallower than 200 m predicts that >50% of the SMLs have a thickness of 0-5 cm, with only 3% thicker than 30 cm (Fig. 3a). The maximum SML thickness of nearly 200 cm was found in the Amazon Delta (Fig. 3b) due to the abundant river particulate sources and high bottom stress. Other areas with thick SMLs include the inner shelf of the Gulf of Mexico (Fig. 3c), and the East China Sea (Fig. 3d). Areas such as the northwest Australia, the Fly River Estuary, the Bay of Bengal, and the Hudson Bay had SML thicknesses of ca. 30 cm. In high latitude areas such as the Arctic, southern regions of Africa, northern America, and southern Australia, SMLs approached 0 cm. Overall, these model simulation results matched well with empirical observations (Fig. 2) with a correlation coefficient of 0.73 (Supplementary Fig. 4).

Based on a probability distribution of modeled average SML thicknesses (Supplementary Fig. 5), the average SML thickness in fine-grained sediments of the global coastal ocean is estimated to be in the range of 7.0 ~ 10.0 cm. Most modeled results occur between 8.0 ~ 9.5 cm, with a mean value of 8.5 ± 0.6 cm, which is between previous estimates of 5.7 cm^33^ and 9.8 cm^32^. The timescale of SML formation varies depending upon the environmental conditions. Individual particles within the SML, both organic and inorganic, will range in age depending upon the sediment accumulation rate and intensity of physical and biological mixing^45^. For example, mudflats adjacent to the Amazon River estuary have deposition rates reaching 1 cm per day^46^. Similarly, continental shelf sediments near the mouth of the Yangtze River, have short-term deposition rates of 4.4 cm per month^47^. In the northern Gulf of Mexico, near the Mississippi/Atchafalaya LDE complex, SMLs with thicknesses of 10–20 cm, were formed within 1 year^48^. Many reported SMLs deposit over seasonal to inter-annual timescales^49^.

Marine sediments host significant levels of biogenic materials that drive biogeochemical exchange, carbon storage and regulation of greenhouse gases^50^. Previous studies addressed mixing intensity and depth in marine muds dominated by bioturbation^33,51,52^. We focus here on coastal ocean sediments, where mixing is strongly affected by physical (e.g., waves and/or currents set up by rivers, storms), biological (e.g., bioturbation) and human (e.g., trawling) driving forces. The presence of hypoxia/anoxia will limit the presence of infauna which, in turn, will limit the thickness of SMLs driven by biological factors. For example, in cases where bottom waters are anoxic, such as the basins off California, SMLs are absent^8^. Hypoxia/anoxia is increasing in many areas around the world such as in the northern Gulf of Mexico^53^, where SML thicknesses are likely to decline with smaller, more opportunistic benthic species dominating in sediments^54^. Our neural network model allows for a more comprehensive and quantitative understanding of reworked muds, induced by physical, biological, and anthropogenic factors, in the coastal ocean.

### Implications for carbon storage

As anthropogenic climate change modifies the global ocean, the importance of CO_2_ sequestration and carbon storage in sediments has received considerable attention^55^. Coastal margins including mangroves and saltmarshes cover ~16% of the global seabed area but account for > 90% of total ocean OC burial^56^, thereby playing a central role in the global carbon cycle. Reworked muds, including mobile muds, resuspended sediments, and bio-mixed muds, are subject to long-term hydrodynamic sorting, which has significant impacts on OC transport, degradation, and deposition on ocean margins, as well as interpretations of related climate records^57^.

Continuous resuspension and redeposition drive periodic oxidation-reduction cycles that accelerate the degradation of organic matter, making the mixed zones effective “incinerators” of organic matter^58^. Enhanced remineralization of sedimentary OC can reduce OC accumulation rates (OCAR)^59^; in contrast, to more quiescent sedimentary environments, that typically favor OC preservation^6^. Total organic carbon/specific surface area (TOC/SSA) ratios, commonly used as an indicator of OC preservation that normalizes grain size effects, are usually lower in mobile-mud deposits and higher in high productivity/upwelling regions^60,61^.

**Fig. 4:**
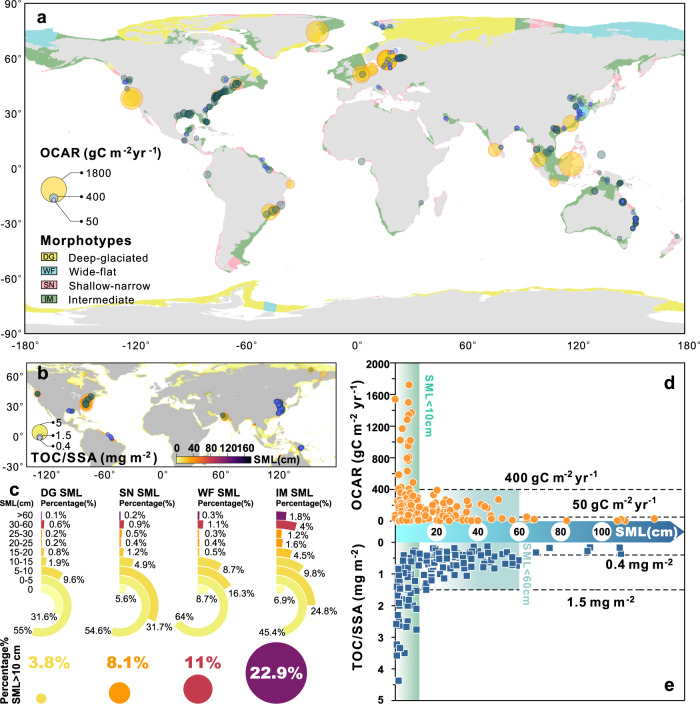
Impacts of sediment mixed layer (SML) on organic carbon storage. **a** Distribution of organic carbon accumulation rates (OCAR) in the global coastal ocean (reproduced with permission from “Harris, P. T. & Macmillan-Lawler, M. Global Overview of Continental Shelf Geomorphology Based on the SRTM30_PLUS 30-Arc Second Database. In: Finkl, C., Makowski, C. (eds) *Seafloor Mapping along Continental Shelves*. 169-190 (2016)”, copyright of © 2016 Springer International Publishing Switzerland); **b** Distribution of the total organic carbon/specific surface area (TOC/SSA) ratios in global coastal sediments; **c** Sediment mixed layer (SML) in different shelf morphotypes; **d** Plot of OCAR versus SML thickness in global coastal ocean sediments; **e** Plot of OCAR and TOC/SSA ratios versus SML thickness in global coastal ocean sediments. Maps generated with python3-mpltoolkits.basemap (version 1.2.1, https://matplotlib.org/basemap/).

Sediment mixing, as reflected by thicknesses of SMLs, appears to strongly impact organic carbon (OC) preservation in the coastal ocean. The distribution patterns of OCAR (Fig. 4a) and TOC/SSA ratios (Fig. 4b) indicate that OC preservation in different geomorphic settings is highly related to SML thickness. The global coastal margins can be divided into four major morphotypes: narrow-shallow type (SN), deep-glaciated type (DG), wide-flat type (WF), and shelves having intermediate values to the other three morphotypes (IM)^62^. The average thicknesses of SMLs, in these four morphotypes, are 5.8, 3.6, 6.5 and 9.9 cm, respectively. Intense sediment reworking is mostly found in IM coastal-shelf settings, with >22% of SML deeper than 10 cm and about 2% >60 cm (Fig. 4c). Most of the largest, river-dominated coastal margins (e.g., Amazon, Mississippi, and Ganges) are categorized as the IM type (Fig. 4a). An inverse relationship between OCAR and SML thickness (Fig. 4d) implies that OCAR decreases significantly when the SML thickness is thicker than 10 cm.

For regions with OCAR >400 g C m^−2^ yr^−1^, almost all SMLs are thinner than 10 cm. For example, the mangrove-dominated Indonesian coast has OCAR = 1722 g C m^−2^ yr^−1^ with an SML of <10 cm (Fig. 4a). In contrast, the SML in the Amazon Shelf is thicker than 60 cm and OCAR is <50 g C m^−2^ yr^−1^. In the mobile-mud belt of the Amazon Estuary, >50% of the OC input from the river is oxidized and decomposed, and only 13–17% of the overall OC input is stored in the seabed^14^. Frequent resuspension and redeposition of mobile muds enhance OC degradation by increasing exposure to powerful oxidants such as oxygen^14^, as well as labile organic matter that acts as primers^47^. Mobile muds are thus important sites for remineralization of OC. Similarly, high TOC/SSA ratios are found at sites with thin SML (e.g., >1.5 mg m^−2^ when SMLs <10 cm), and lower TOC/SSA ratios in areas with thick SMLs (e.g., <0.4 mg m^−2^ when SMLs >60 cm) (Fig. 4e).

In the other three coastal morphotypes, <10% of SMLs have thicknesses >10 cm (Fig. 4c). In the DG coastal-shelf settings, >30% of the sites have negligible SMLs. At high latitudes, deglaciation is changing coastal sedimentary dynamics, particularly in fjords^63^, resulting in local changes in the density of deposits and sources of OC^64^. Weak sediment reworking is associated with OC preservation in polar continental margins. For example, coastal sediments in north-east Greenland, characterized by sediments with shallow SMLs had OCAR at 1540 g C m^−2^ yr^−1^ (Fig. 4a)^65^. Therefore, identifying the thickness and distribution of SMLs in high latitude systems needs further attention, as these regions experience dramatic alterations in the sedimentary, cryospheric and hydrologic cycles (https://www.ipcc.ch/report/sixth-assessment-report-working-group-i/).

Narrow shelf (SN) coastal oceans minimize mineralization of carbon along the transmission path^66^. Interestingly, reported TOC/SSA ratios are all >0.4 mg m^−2^ (Fig. 4b), consistent with more rapid transport and reduced OC decay in such geomorphic settings. Wider shelves (WF) with widths up to 380 km largely occur in high latitude regions such as the Arctic, the Siberian Shelf and Chukchi Sea (Fig. 3a) and had very thin SMLs (<5 cm). Lower latitude WF settings (e.g., Yellow Sea and the inner shelf muddy area of the East China Sea^60^) have thicker SMLs. Sedimentary OC in these mobile-mud regions experience frequent sediment reworking with rapid Fe redox cycling and long-distance transport, resulting in low OC burial (Fig. 4a, b) and OC preservation efficiency (~30%)^67–69^.

The estimated thicknesses of SMLs here are based on a decadal time scale from ^210^Pb profiles, provide a strong connection for assessing carbon storage. These correlations provide global-scale evidence that refreshed exposure of sedimentary OC to the overlying water column reduces OC accumulation. Specific mechanisms will vary among sites and will have different impacts among shelf morphotypes. The simulation presented here provides a first step establishing a more global characterization of SMLs in the current global coastal ocean. This characterization can lead to more insights about hotspots of organic matter cycling in marine sediments^56^, which can more broadly support Earth System Models. Finally, as many marine macrofaunal benthos in the coastal ocean are undergoing poleward range expansion due to global warming^70^, SMLs will likely undergo additional change that will also need to be included in ongoing modeling efforts.

## Methods

### Data source

The Web of Science (Thomson Reuters, New York, NY), Google Scholar, and Bai Du Scholar were utilized to search the literature using the following key words or phrases: ^210^Pb with coastal and/or estuary; and reworked muds and/or mixed layer. The data repository created by Solan et al.^71^ provided an excellent base to show relationships between benthic faunal community distribution and sediment characteristics, and we also obtained additional ^210^Pb_ex_ profiles from references therein. We compiled 238 studies with 1093 sites having ^210^Pb_ex_ profiles distributed globally. Data source references are presented in the Supplementary Data 1. About 62% of the sites were located on continental shelves between 0 ~ 200 m water depth, and the remaining locations were in intertidal mud/sand flats, wetlands, and the deep ocean.

Environmental factors, physical dynamics and biological activities were collected for each site and presented in Supplementary Data 1, including mean annual precipitation, water depth, total suspended matter, tidal range, relative sea level rise rate, tropical cyclone frequency, bottom stress, primary productivity, river discharge, river sediment load and sediment accumulation rate, to explore for drivers of the SML in the coastal zone.

Tidal ranges were extracted from a global tidal range dataset^72,73^ and water depths from the ocean bathymetry database (https://www.ngdc.noaa.gov/mgg/global/global.html). Tropical cyclone effects and hazard risks were based on the Global Cyclone Hazard Frequency and Distribution, v1 dataset (1980–2000), and assessed on a 2.5 min’ global grid. More than 1600 storm tracks were assembled and modeled, through the period January 1st, 1980, to December 31st, 2000, for the Atlantic, Pacific, and Indian Oceans, at UNEP/GRID-Geneva PreView (https://www.ldeo.columbia.edu/chrr/). Sediment types were collected from seafloor lithology in the GplatesPortal^74^. We added about 7800 points in areas with poor data coverage and reproduced a new digital map of seafloor lithology (Supplementary Fig. 6). We simulated SML only for sites with fine-grained sediment (silt and clay). In coastal wetlands, sediment accumulation rates were obtained from a recent global assessment of saltmarshes and mangroves^75^. Data of mean annual precipitation were acquired from world climate data, European Climate Assessment & Dataset^76^. For relative sea level rise rates, data were collected from the Permanent Service for Mean Sea Level database^77^. Total suspended matter was derived from Medium Resolution Imaging Spectrometer satellite data, processed in the framework of the GlobColour project^73^. The dataset of river discharge comes from “Dai and Trenberth Global River Flow and Continental Discharge Dataset”^78^, which were interpolated according to the river discharge and distance to obtain the river discharge influence at each data point. The data of bottom currents are from TPXO global tide models^79^. The sediment load of global large rivers is from the dataset by Milliman and Katherine^80^. Satellite-observed monthly global climatology sea surface chlorophyll-a concentration and primary production with 4 km resolution were downloaded from Copernicus Marine Service (https://resources.marine.copernicus.eu/). Annual average chlorophyll-a concentration and primary production were originally calculated using these climatology data, which were then linearly interpolated for each location. Part (165 sites) of the organic carbon accumulation rates (OCAR) data for global tidal marshes and mangroves was extracted from the study of Wang et al.^75^. The remaining estimates of OCAR and TOC/SSA ratios were collected from the literature and both the actual data and relevant references are listed in the Supplementary Data 1.

### The neural network model

A supervised multilayer perceptron (MLP), the most commonly applied type of neural networks^81^, was employed to assess the distribution of SML in the global coastal ocean. Briefly, a neural network is a set of neurons that can be connected and combined in one or multiple layers. The first layer, called the input layer, consists of the source data. There are then intermediate layers, called hidden layers. The resulting output, in this case the thickness of the SML, is obtained in the last layer. Two key issues in MLP design are the specification of the number of hidden layers and the number of neurons in these layers. Once the number of layers and number of neurons in each layer have been selected, the network’s weights and thresholds must be set to minimize the prediction error made by the network; this task is the role of the training algorithms.

Four essential steps were used in designing the neural network: (a) collecting data, (b) preprocessing data, (c) building the network, and (d) training and test performance of the model (Supplementary Fig. 7)^82^. After data collection, correlation analysis and data normalization were used to train the neural network more efficiently. For correlation analysis, several highly correlated factors were selected, and transformed into values between 0 and 1 using data normalization. The selected data were divided into two randomly selected groups, the training group which corresponded to 70% of the patterns, and the test group, which corresponded to the remaining 30% of the patterns.

Training a MLP helps to accurately estimate desired dependent variables or outputs. To do this, multiple calculations are carried out to modify the weights of every one of the connections between neurons. The first step in this process is achieved with an activation function (equation [1]), which handles all the data that enters a neuron^83^.

1 $${x}_{k}=\mathop{\sum}\limits_{j=1}{w}_{{jk}}{y}_{j}$$

where *w*_*jk*_ stands for the weight that represents the connection between layers *j* and *k*, *y*_*j*_ is the value of the input, which is introduced in a neuron, and *x*_*k*_ is the solution provided by the activation function.

The accuracy of the various predictions was evaluated using the correlation coefficient (CC), the root mean-square error (RMSE), the mean absolute error (MAE), and percent correct (PC) between the measured values and the predicted values. Here, we used MAE and R^2^ score to evaluate the neural network model. There are other algorithms with very different principles that may also be suitable for predicting SMLs, such as K-nearest neighbor (KNN), Random Forest (RF) and support vector machine (SVM). We compared each algorithm based on their performance and found that the MLP was the most applicable algorithm for predicting global SML (Table S1).

### Structure of the neural network

The optimal architecture of the developed artificial neural network revealed three hidden layers of 20, 40 and 10 neurons (Supplementary Fig. 8). The neurons in the input layer were equal to the five predictors with the best statistical correlation results. This nonlinearity in a neural network model presents advantages and disadvantages. For example, it is difficult to determine the values of the parameters in a computationally intensive nonlinear optimization. Thus, a trial-and-error approach was used to determine a network with optimum performance. After each iteration, the network outputs were compared to the actual target values until the performance function was maximized.

Output values were compared with expected values based on the training data, and the errors computed. Through iterative propagation of errors back to the network, the connection weights were automatically adjusted until the target minimum error was attained. To achieve this, different tests were conducted, and the best learning rate and training time (epoch) obtained were constant at a level of 3000. When epochs reached 3000, the MAE decreased to 0.32 (after data normalization) and remained stable, and the R^2^ was 0.475.

### Global upscaling based on the neural network model

A global map was equally divided into 1000 × 1000 grids using ArcGis (10.2), resulting in about 50,000 sites along the coastal zone and continental shelf to water depths of 200 m. Each grid is 20 × 10 arcmin with an area of ca. 510 km^2^. The operational steps in ArcGis included attributes extraction, fishnet creation, location selection, and data export. Finally, we entered the grid into the trained neural network model to predict the thickness of SML with the same grid resolution. The model prediction results are available as a downloadable file (Supplementary Data 2).

## Supplementary information

Supplementary Information

Description of Additional Supplementary Files

Supplementary Data 1

Supplementary Data 2

## Data Availability

The data generated in this study are provided in the Supplementary Information and Supplementary Data 1 and 2, and also deposited in the Zenodo online repository at 10.5281/zenodo.6901752.

## Code availability

The code used in this study are available at: 10.5281/zenodo.6890194.
