# Supplementary material for: A global assessment of the mixed layer in coastal sediments and implications for carbon storage
Source: Nat Commun. 2022 Aug 20;13:4903. doi: 10.1038/s41467-022-32650-0 (PMC9392783; doi:10.1038/s41467-022-32650-0)
Supplement: Supplementary file 1 — Supplementary Information [file 41467_2022_32650_MOESM1_ESM.pdf]

Supplementary information for:

A global assessment of the mixed layer in coastal sediments and implications for carbon storage

Shasha Song[1,2,3], Isaac R. Santos[4,5], Huaming Yu[6,7], Faming Wang[8], William C. Burnett[9], Thomas S. Bianchi[10], Junyu Dong[11], Ergang Lian[12], Bin Zhao[1,2], Lawrence Mayer[13], Qingzhen Yao[1,2], Zhigang Yu[1,2], Bochao Xu[1,2*]

[1] Frontiers Science Center for Deep Ocean Multispheres and Earth System, Key Laboratory of Marine Chemistry Theory and Technology, Ministry of Education, Ocean University of China, 266100 Qingdao, P.R. China

[2] Laboratory for Marine Ecology and Environmental Science, Qingdao National Laboratory for Marine Science and Technology, 266100 Qingdao, P.R. China

[3] College of Chemistry and Chemical Engineering, Ocean University of China, 266100 Qingdao, P.R. China

[4] Department of Marine Sciences, University of Gothenburg, Gothenburg, Sweden

[5] National Marine Science Centre, School of Environment, Science and Engineering, Southern Cross University, Coffs Harbour, 2450 NSW, Australia

[6] College of Oceanic and Atmospheric Sciences, Ocean University of China, 266100 Qingdao, P.R. China

[7] Sanya Oceanographic Institution, Ocean University of China, 572000 Sanya, P.R. China

[8] Xiaoliang Research Station for Tropical Coastal Ecosystems, Key Laboratory of Vegetation Restoration and Management of Degraded Ecosystems, and the CAS engineering Laboratory for Ecological Restoration of Island and Coastal Ecosystems, South China Botanical Garden, Chinese Academy of Sciences, 510650 Guangzhou, P.R. China

[9] Department of Earth, Ocean, and Atmospheric Science, Florida State University, Tallahassee, 32306 Florida, USA

[10] Department of Geological Sciences, University of Florida, Gainesville, 32611-2120 Florida, USA

[11] School of Computer Science and Technology, Ocean University of China, Qingdao, 266100 P.R. China

[12] State Key Laboratory of Marine Geology, Tongji University, 200092 Shanghai, P.R. China

[13] School of Marine Sciences, University of Maine, Walpole, Maine 04573, USA

This supplementary document contains the following information:

Supplementary Discussion 1 to 3

Supplementary Methods 1 to 3

Supplementary Fig. 1 to 8

Supplementary Table 1

Supplementary Discussion 1: Global distribution of sediment mixed layer

Global distribution of SMLs (Supplementary Fig. 1). SMLs in most area are thinner than 5 cm. Approximately 20% of the cores had no SML, 27% had SMLs that ranged from 0 to 5 cm, and 3% had SMLs >30 cm. There are significant differences among continents, especially between the Americas and Asia. The majority of SMLs in the Americas have thicknesses of 0-5 cm. In Asia, many locations have SMLs with thicknesses of 10-20 cm, and in some cases > 30 cm, partly because Asia has the largest number of great rivers, many with high sediment loads[43].

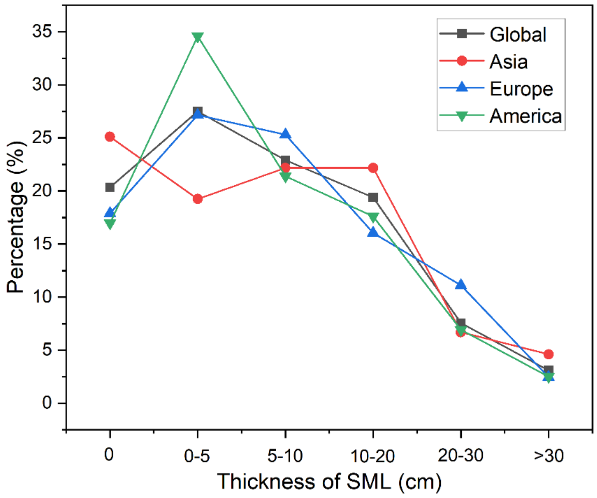

Supplementary Fig. 1. Global distribution of thicknesses of sediment mixed layer (SML) and their relative percentages.

Supplementary Methods 1: Data collection

We collected a total of 12 factors that may influence the formation of SML: water depth, mean annual precipitation, relative sea level rise rate, tropical cyclone frequency, mean tidal range, sediment accumulation rate, total suspended matter, chlorophyll-a concentration, primary productivity, bottom stress, river sediment load and river discharge. The individual Pearson correlation coefficients between these factors and SML are shown in the Supplementary Fig. 2 below. The raw data of all input parameters are shown in Supplementary Data 1.

Here we present a detailed data development for bottom stress and river discharge. Tidal currents and wind waves are the dominant driving forces for sediment re-suspension and transport in the bottom boundary layer of shallow seas. In general, the shear stress is calculated using the approximate formula $\tau=\rho C\text{-}d\ U^2$, $C\text{-}d \approx 3\times10^{-3}$ is seabed friction coefficient[1]. The tidal current speed is from TPXOv9 calculated from satellite altimeters[2]. The bottom orbital velocity beneath waves is calculated using the ECMWF Reanalysis v5 (ERA5) dataset[3].

We obtained multi-year mean observed river discharge data of global major rivers from the dataset by Milliman[4]. Then the global annual mean ocean circulation is calculated using the HYCOM high-resolution reanalysis ocean current dataset GOFS 3.1[5-7]. The convection diffusion equation is used to calculated the river diluted water spread using the discharge data and circulation data.

$$\frac{\partial \mathbf{C}}{\partial \mathbf{t}} + \mathbf{u}\frac{\partial \mathbf{C}}{\partial \mathbf{x}} + \mathbf{v}\frac{\partial \mathbf{C}}{\partial \mathbf{y}} + \mathbf{w}\frac{\partial \mathbf{C}}{\partial \mathbf{z}} = \mathbf{K_h}\left(\frac{\partial^2 \mathbf{C}}{\partial \mathbf{x}^2} + \frac{\partial^2 \mathbf{C}}{\partial \mathbf{y}^2}\right) + \mathbf{K_z}\frac{\partial^2 \mathbf{C}}{\partial \mathbf{z}^2} + \mathbf{C_0}(x, y, z, t)$$

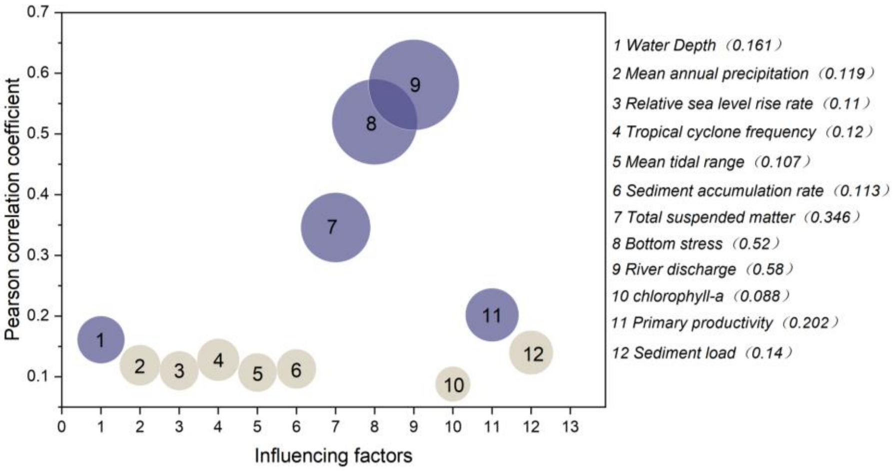

Supplementary Fig. 2. The relationship between 12 influencing factors and thickness of sediment mixed layer (SML). The proposed five variables that best controlled SMLs thickness, marked in purple, were used to train the neural network model.

Supplementary Discussion 2: Influencing factors of sediment mixed layer

Plots of simulated SML thicknesses vs. bottom stress and primary production (Supplementary Fig. 3 below), illustrate the relative importance of physical and biological driving forces in different regions. The highest SMLs were linked with the highest bottom stress in large river estuaries, such as the Amazon estuary, which is about 120,000 Pa with SMLs thicknesses of almost 2 m. Thickness of SMLs also increased in regions with high primary productivity (3000 - 5000 mg/m^2/day) and weak physical forces (e.g., bottom stress of 0-1000Pa), such as the coast of north Colombia and Venezuela, which SML thickness is usually around 30 cm.

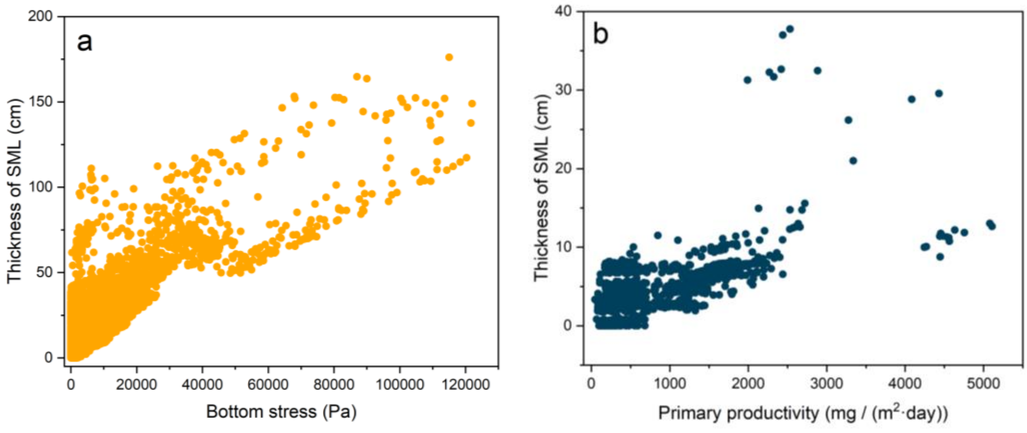

Supplementary Fig. 3. Relationship between physical, biological forces and sediment mixed layer (SML). (a) Plot of SML thickness versus bottom stress; (b) Plot of SML thickness versus primary productivity with bottom stress <1 Pa.

Plot of measured SMLs and predicted SMLs (Supplementary Fig. 4), showing a significant linear correlation relationship (0.73), which further corroborates our model evaluation metrics.

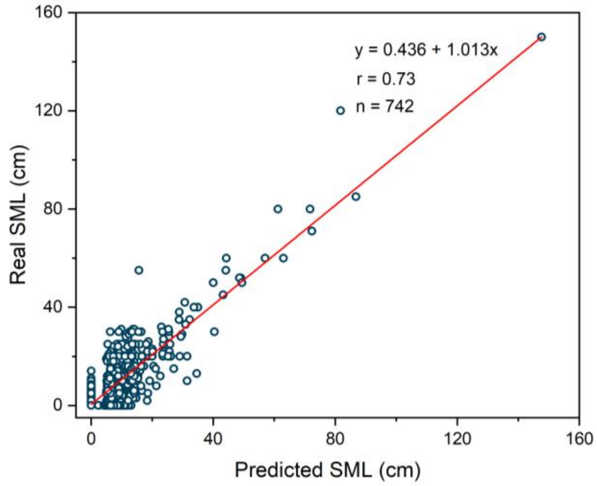

Supplementary Fig. 4. The relationship of measured sediment mixed layers (SML) (from measured $^{210}Pb_{ex}$ profiles) and predicted SMLs.

Supplementary Methods 2: Average sediment mixed layer

We conducted 100 global simulations and obtained a global average SML thickness each time. From these simulations, a probability distribution diagram was made (Supplementary Fig. 5), with an associated mean value and standard error.

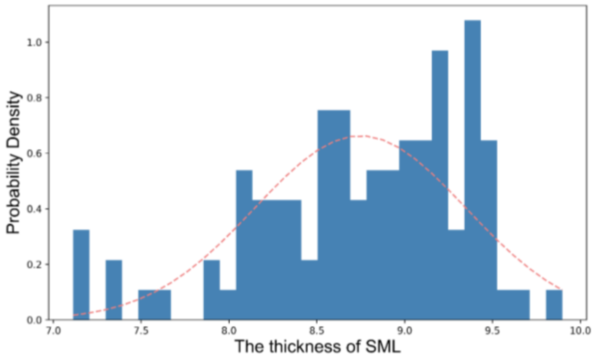

Supplementary Fig. 5. Probability distribution of the modeled average thickness of sediment mixed layer (SML) which ranged from 7~10 cm; this result is derived from 100 simulations in our model.

Supplementary Discussion 3: Sediments in global ocean

An additional 7800 data points were added in regions with poor data coverage, such as the English Channel, Hudson Bay and the China coastal seas. This was used to develop a new digital map of sediments-type distribution in the global ocean, based on the method of Dutkiewicz et al.[8](Supplementary Fig. 6).

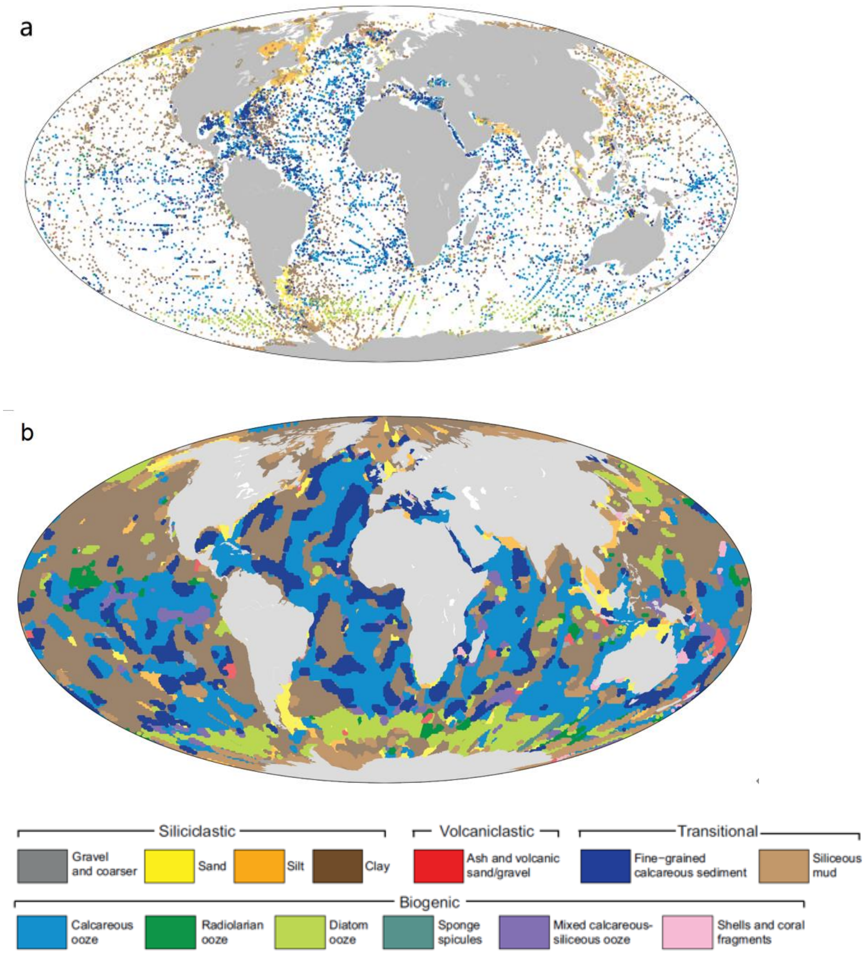

Supplementary Fig. 6. Grain size of sediments in the global ocean. (a) Seafloor sediment sample locations with 14399 original data points[8] (labeled as solid dots) and 7800 data points complied from other sources (labeled as solid stars, references and data are presented in Supplementary Data 1). (b) Digital map of major lithologies of seafloor sediments in the world ocean basins. These two figures were reproduced with permission from “Dutkiewicz, A., Müller R.D., O'Callaghan, S. & Jóson, H. Census of seafloor sediments in the world’s ocean. Geology. 43, 795-798 (2015), copyright of © 2015 Geological Society of America.

Supplementary Methods 3: The Neural Network model

MAE (equation [1]) and R^2 score (equation [2]) were used to evaluate the neural network model. The smaller the MAE and the larger the R^2, the better the model.

$$\mathbf{MAE} = \frac{\sum_{i=1}^{n} |y_i - x_i|}{n} \qquad [1]$$

Where n stands for the number of data points, y_i is the target value, and $x_i$is the predicted value.

$$\mathbf{R^2} = 1 - \frac{\sum_{i=1}^{n}(x_i - y_i)^2}{\sum_{i=1}^{n}(x_i - p_i)^2} \qquad [2]$$

Where n stands for the number of data points, y_i is the target value, x_i is the predicted value, and p_i is average of target values.

Five algorithms were chosen to simulate the global SML, including K-nearest neighbor (KNN), support vector machine (SVM), random forest (RF), gradient boosting regression (GBR) and multilayer perceptron (MLP); this was used to train and test our data respectively. These algorithms have very different principles and applicable fields. Through the comparison of these results (Table 1), the MLP is the most suitable algorithm for the prediction of our data.

Supplementary Table 1. A comparison of multiple algorithms to assess the most effective when modelling our data. MAE (mean absolute error) and R^2 score of K-nearest neighbor (KNN), support vector machine (SVM), Random Forest (RF), Gradient boosting regression (GBR) and MLP respectively.

	KNN	SVR	RF	GBR	MLP
MAE	6.90	6.83	6.47	7.095	6.53
R^2	0.215	0.03	0.285	0.32	0.475

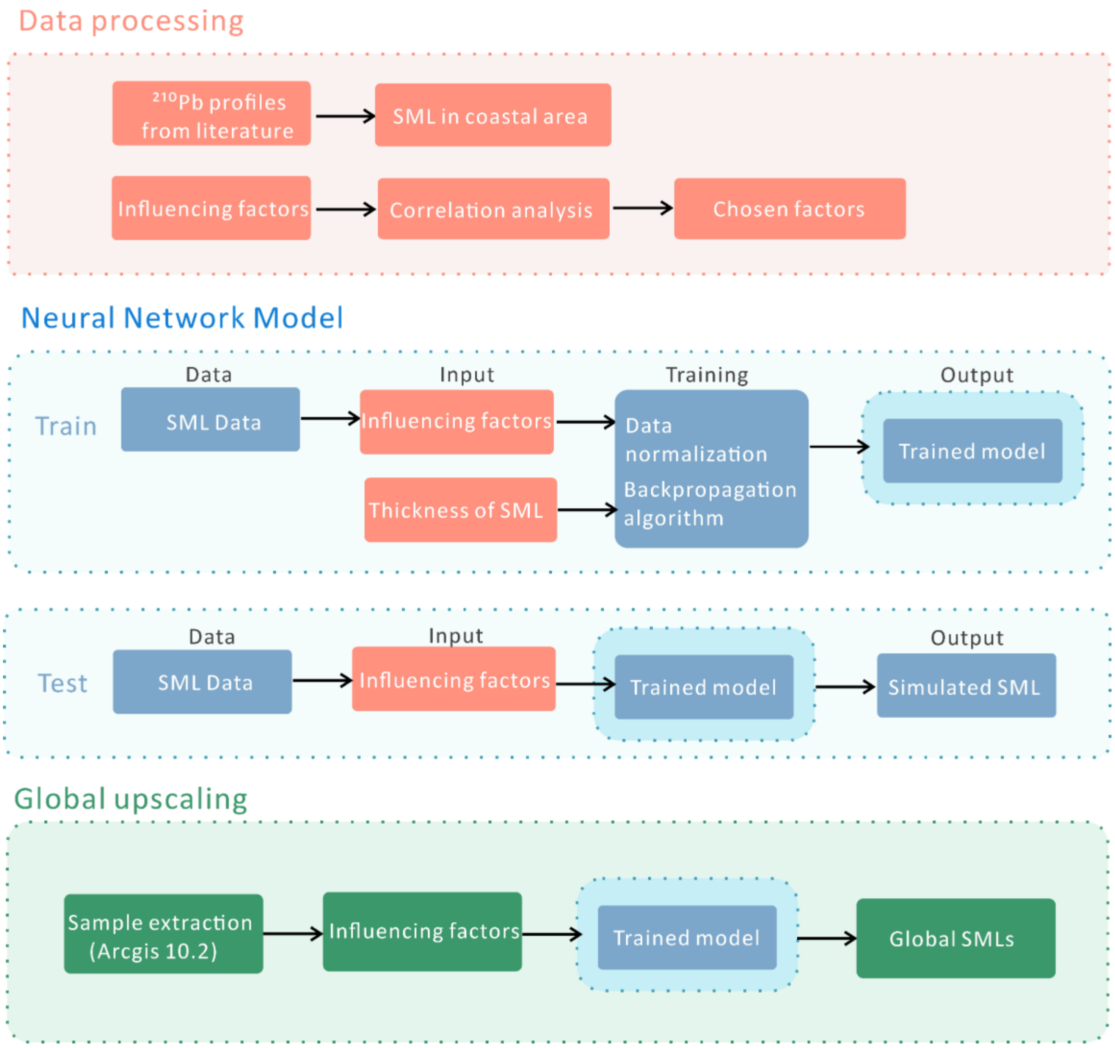

Supplementary Fig. 7. Summary of the methods used in neural network construction of SMLs in the coastal ocean. Orange represents the data processing, including data collection and correlation analysis; Blue is shown for implementation of the neural network model; and Green is used for the global upscaling of SML in coastal zone.

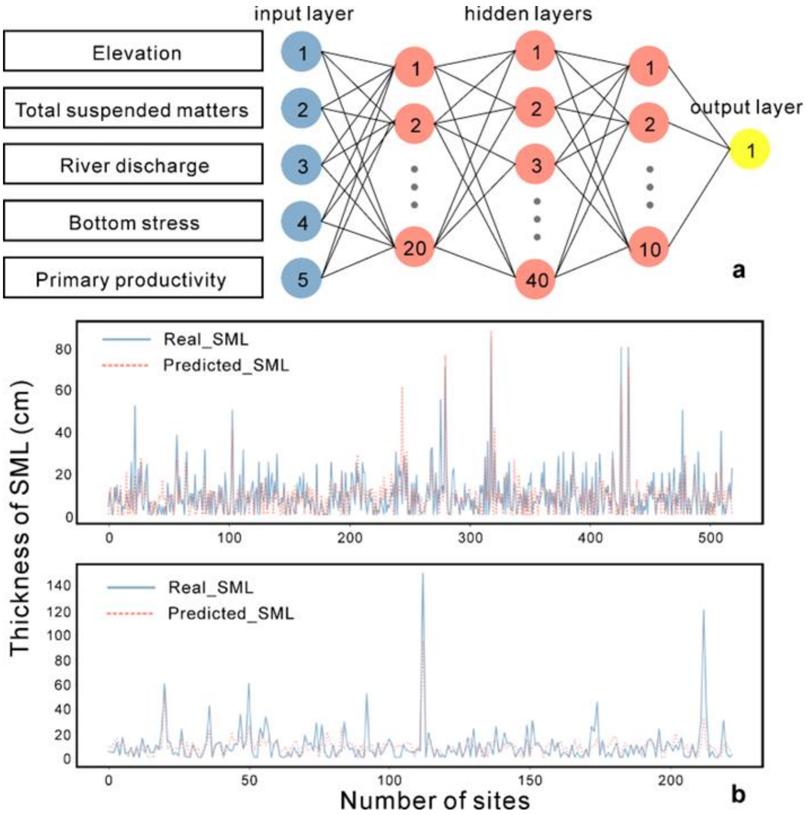

Supplementary Fig. 8. Sketch of the neural network structure used for construction of SMLs in the coastal ocean (a). The model consists of input layer (blue), 3 hidden layers (orange) and output layer (yellow). The input layer includes five features: water depth, total suspended matter, river discharge, bottom stress and primary productivity. The output layer is the predicted SML. Three hidden layers have 20, 40 and 10 neurons respectively. (b) Plots of measured SML and predicted SMLs. The data were divided into a training set (upper panel) and testing set (lower panel).

Supplementary References

1. Shi, B. W., Yang, S. L., Wang, Y. P., Bouma, T. J. & Zhu, Q. Relating accretion and erosion at an exposed tidal wetland to the bottom shear stress of combined current-wave action. *Geomorphology* **138**, 380-389 (2012).

2. Egbert, G. D. & Erofeeva, S. Y. Efficient inverse modeling of barotropic ocean tides. *J. Atmos. Oceanic Technol.* **19**, 183-204 (2002).

3. Wiberg, P. L. & Sherwood, C. R. Calculating wave-generated bottom orbital velocities from surface-wave parameters. *Comput. Geosci*. **34**, 1243-1262 (2008).

4. Milliman, J. D. & Katherine L. F. River discharge to the coastal ocean: a global synthesis. *Cambridge University Press*, 2013.

5. Cummings, J.A. Operational multivariate ocean data assimilation. *Quart. J. Royal Met. Soc., Part C,* **131**, 3583-3604 (2005).

6. Cummings, J. A. & Smedstad, O. M. Variational data assimilation for the global ocean. *In Data Assimilation for Atmospheric, Oceanic and Hydrologic Applications* **Vol. II**, 303-343 (2013).

7. Helber, R. W., Townsend, T. L., Barron, C. N., Dastugue, J. M. & Carnes, M. R. Validation test report for the Improved Synthetic Ocean Profile (ISOP) system, Part I: Synthetic profile methods and algorithm. *Naval Research Lab Stennis Detachment Stennis Space Center Ms Oceanography Div* (2013).

8. Dutkiewicz, A., Müller R.D., O'Callaghan, S. & Jóson, H. Census of seafloor sediments in the world's ocean. *Geology*. **43**, 795-798 (2015).
