# Supplementary material for: A global assessment of the mixed layer in coastal sediments and implications for carbon storage
Source: Nat Commun. 2022 Aug 20;13:4903. doi: 10.1038/s41467-022-32650-0 (PMC9392783; doi:10.1038/s41467-022-32650-0)
Supplement: Supplementary file 2 — Description of Additional Supplementary Files [file 41467_2022_32650_MOESM2_ESM.pdf]

File name: Supplementary Data 1

Description: Input features of model, $^{210}Pb_{ex}$ profiles and carbon data from the literature

File name: Supplementary Data 2

Description: Simulated SMLs in the global coastal ocean
